# Effect of Cancer on Clinical Outcomes of Patients With COVID-19: A Meta-Analysis of Patient Data

**DOI:** 10.1200/GO.20.00225

**Published:** 2020-06-08

**Authors:** Vassilis G. Giannakoulis, Eleni Papoutsi, Ilias I. Siempos

**Affiliations:** ^1^ First Department of Critical Care Medicine and Pulmonary Services, Evangelismos Hospital, National and Kapodistrian University of Athens Medical School, Athens, Greece; ^2^ Division of Pulmonary and Critical Care Medicine, Department of Medicine, New York-Presbyterian Hospital–Weill Cornell Medical Center, Weill Cornell Medicine, New York, NY

## Abstract

**PURPOSE:**

Whether cancer is associated with worse prognosis among patients with COVID-19 is unknown. We aimed to quantify the effect (if any) of the presence as opposed to absence of cancer on important clinical outcomes of patients with COVID-19 by carrying out a systematic review and meta-analysis.

**METHODS:**

We systematically searched PubMed, medRxiv, COVID-19 Open Research Dataset (CORD-19), and references of relevant articles up to April 27, 2020, to identify observational studies comparing patients with versus without cancer infected with COVID-19 and to report on mortality and/or need for admission to the intensive care unit (ICU). We calculated pooled risk ratios (RR) and 95% CIs with a random-effects model. The meta-analysis was registered with PROSPERO (CRD42020181531).

**RESULTS:**

A total of 32 studies involving 46,499 patients (1,776 patients with cancer) with COVID-19 from Asia, Europe, and the United States were included. All-cause mortality was higher in patients with versus those without cancer (2,034 deaths; RR, 1.66; 95% CI, 1.33 to 2.07; *P* < .0001; 8 studies with 37,807 patients). The need for ICU admission was also more likely in patients with versus without cancer (3,220 events; RR, 1.56; 95% CI, 1.31 to 1.87; *P* < .0001; 26 studies with 15,375 patients). However, in a prespecified subgroup analysis of patients > 65 years of age, all-cause mortality was comparable between those with versus without cancer (915 deaths; RR, 1.06; 95% CI, 0.79 to 1.41; *P* = .71; 8 studies with 5,438 patients).

**CONCLUSION:**

The synthesized evidence suggests that cancer is associated with worse clinical outcomes among patients with COVID-19. However, elderly patients with cancer may not be at increased risk of death when infected with COVID-19. These findings may inform discussions of clinicians with patients about prognosis and may guide health policies.

## INTRODUCTION

An ever-increasing number of people in the global population are suffering from cancer.^1^ Patients with cancer are therefore anticipated to be affected during the current epidemic of COVID-19. However, whether, when infected with COVID-19, patients with versus without cancer are at increased risk for unfavorable clinical outcomes is unknown. This was highlighted in a plenary session at the American Association for Cancer Research Virtual Annual Meeting held on April 27-28, which subsequently issued a call for relevant research.^2^

**CONTEXT**

**Key Objective**
To quantify the effect (if any) of the presence as opposed to absence of cancer on important clinical outcomes of patients with COVID-19.
**Knowledge Generated**
Cancer is associated with worse clinical outcomes among patients with COVID-19. However, elderly patients with cancer may not be at increased risk of death when infected with COVID-19.
**Relevance**
The findings of the meta-analysis may inform discussions of clinicians with patients about prognosis and may guide health policies.

We therefore aimed to quantify the effect (if any) of the presence as opposed to absence of cancer on important clinical outcomes, such as mortality and need for admission in the intensive care unit (ICU), of patients with COVID-19 by carrying out a systematic review and meta-analysis.

## METHODS

We reported the current systematic review and meta-analysis in accordance with the Preferred Reporting Items for Systematic Reviews and Meta-Analyses statement.^3^ We prespecified inclusion criteria, methods of data synthesis, and outcomes in a protocol registered in PROSPERO (CRD42020181531) and available online

### Eligibility Criteria

We considered observational cohort studies of COVID-19, which reported on all-cause mortality and/or need for ICU admission of patients with cancer versus patients without cancer. Details on the assessment of need for ICU admission are provided in the Data Supplement. Both peer-reviewed papers and preprints were considered, because of the need for use of rapidly accumulated information during the current situation. Reports on coronavirus-caused diseases other than COVID-19 were excluded.

### Search Strategy

We systematically searched PubMed, medRxiv, and CORD-19 (COVID-19 Open Research Dataset). The latter is probably the most extensive machine-readable literature collection specially created for the COVID-19 global crisis. We retrieved all relevant English literature from January 1, 2020, up to April 27, 2020. We also searched references of initially retrieved articles. We used Boolean logic to create the search key phrase (“clinical characteristics” OR comorbidities OR cancer OR malignancy) AND (COVID-19 OR 2019-nCoV OR SARS-CoV-2) AND (mortality OR morbidity OR severity OR ICU OR outcomes). When searching CORD-19, we replaced Boolean operators “AND” and “OR” with the symbols “+” and “|”, respectively. When searching medRxiv, we used “COVID-19 cancer” as the main key phrase. Two authors (V.G.G. and E.P.) independently conducted the literature search and uploaded their findings in an online file storage service (Google Drive) to double-check them. They subsequently discussed the possibility of duplicate patient populations with the third author (I.I.S.).^4^

### Data Extraction and Risk of Bias Assessment

Two authors (V.G.G. and E.P.) independently extracted data in a prespecified worksheet and cross-checked their findings. We collected data on type of publication, author, type of study, total patient population, outcomes of patients with versus without cancer, age, sex, and comorbidities. Authors of original contributions were contacted. Six authors provided us with additional information, which was incorporated in the findings of the meta-analysis.

We assessed the methodological quality of the retrieved observational cohort studies with the Tool to Assess Risk of Bias in Cohort Studies, developed by the CLARITY Group at McMaster University.^5^ The tool uses 8 questions, with 4 possible answers in each. Clarifications on the risk-of-bias assessment are provided in the Data Supplement. Two authors (V.G.G. and E.P.) independently assessed the studies. The results were discussed with the third author (I.I.S.).

### Outcomes of the Meta-Analysis

The primary outcomes of the meta-analysis were all-cause mortality and need for ICU admission. The latter outcome included either actual admission to the ICU or severe disease (such as application of invasive mechanical ventilation) that required admission to the ICU, even if the original study did not specify whether such patients were indeed admitted in the ICU (more details are provided in the Data Supplement). We did so because patients with severe disease might occasionally be unable to be admitted to the ICU because of unavailability of enough beds.

### Statistical Analysis

We performed prespecified sensitivity analyses by calculating the pooled risk ratio (RR) of studies with low risk of bias and by excluding each study and recalculating the RR. We attempted prespecified subgroup analyses by age, type of cancer (solid tumor *v* hematologic malignancy), and country, but we were not able to perform the last 2 analyses because of unavailability of relevant data.

We conducted data synthesis using Review Manager 5.3 (RevMan 5.3) by the Cochrane Collaboration.^6^ We expressed pooled dichotomous effect measures as RR with 95% CI. We used a random-effects (DerSimonian and Laird) model. We measured the presence of statistical heterogeneity with *I*^2^, interpreted according to the Cochrane Handbook recommendations^7^; 0%-40%: might not be important; 30%-60%: may represent moderate heterogeneity; 50%-90%: substantial heterogeneity; 75%-100%: considerable heterogeneity.

## RESULTS

**FIG 1:**
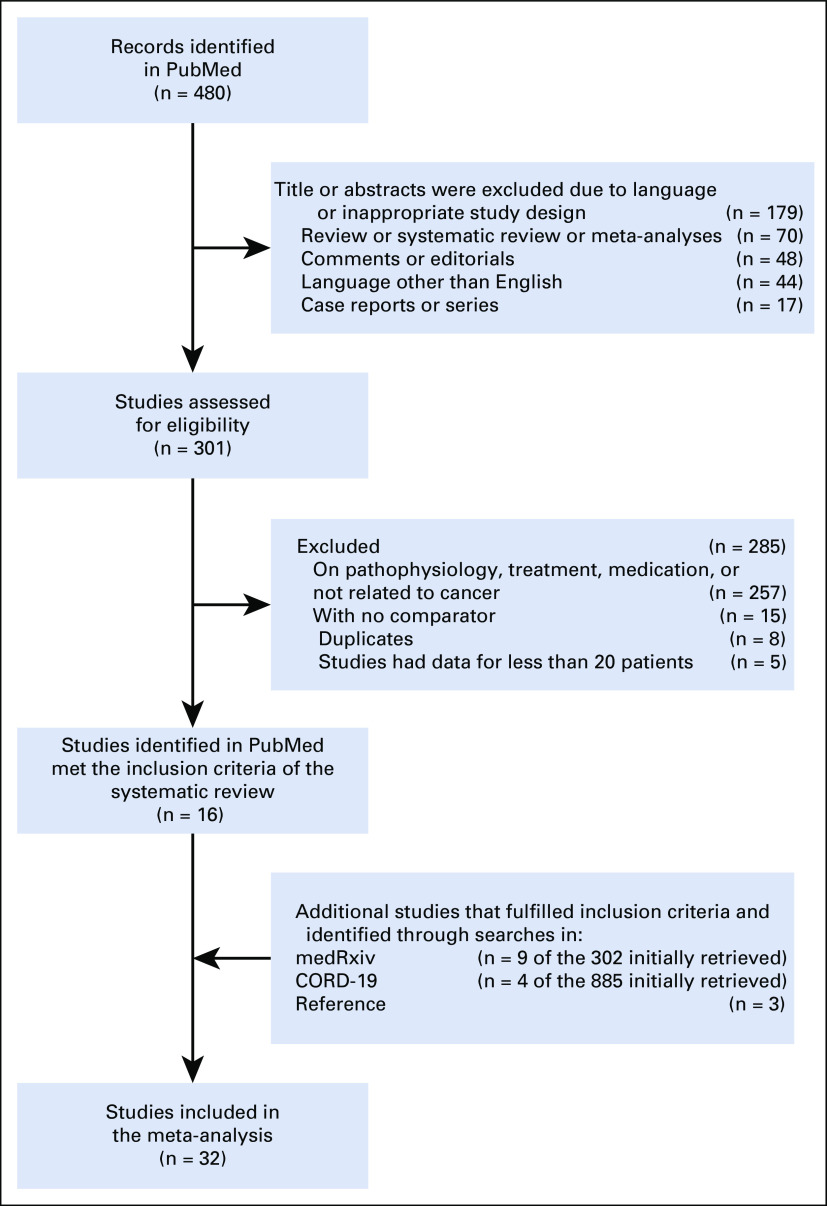
Study flow diagram. CORD-19, COVID-19 Open Research Dataset.

Figure 1 shows the flow diagram for study selection. Regarding mortality data from China, we excluded presumably duplicate publications with overlapping enrollment dates to include only 1 overarching report from the Chinese Center for Disease Control and Prevention (CDC).^8^ However, given that the latter report from the Chinese CDC did not provide specific data on old patients,^8^ and 2 studies from China provided such data,^9,10^ we included these 2 studies in our subgroup analysis by age. A total of 32 studies (19 peer-reviewed, 13 preprints) involving 46,499 patients (1,776 patients with cancer) with COVID-19 from Asia, Europe, and the United States were included in our meta-analysis.^8-39^ Tables 1 and 2 list the summary characteristics and risk of bias assessment of the included studies, respectively.

**TABLE 1:**
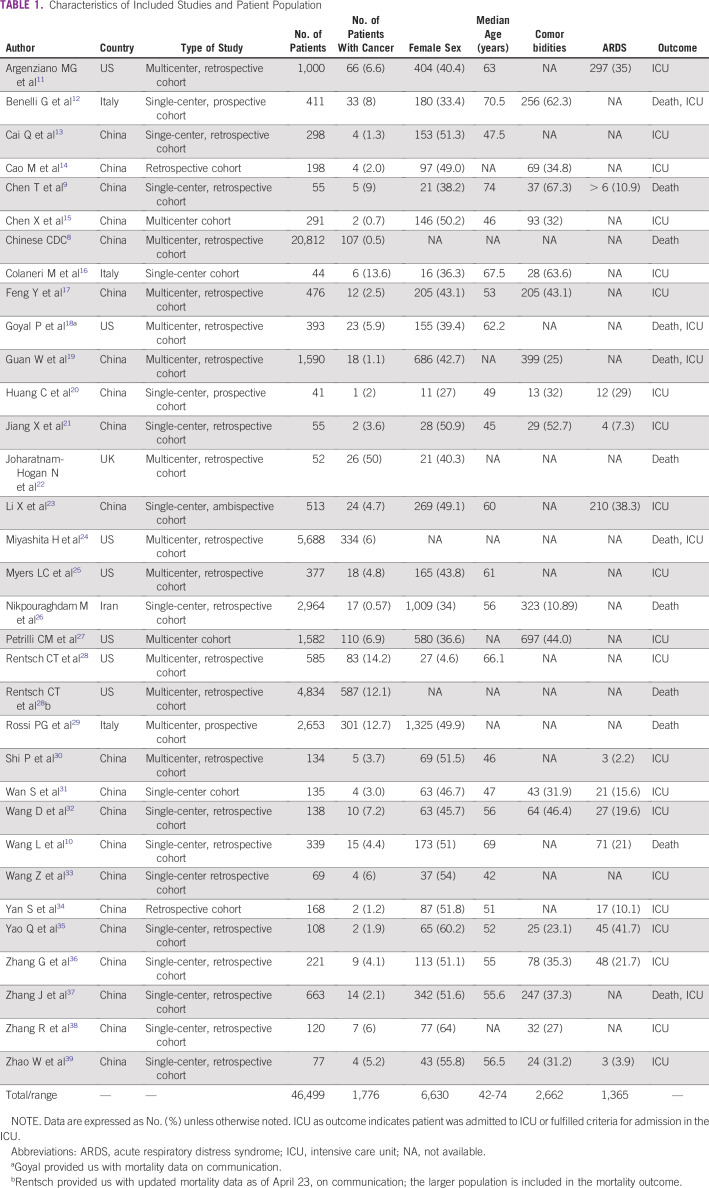
Characteristics of Included Studies and Patient Population

**TABLE 2:**
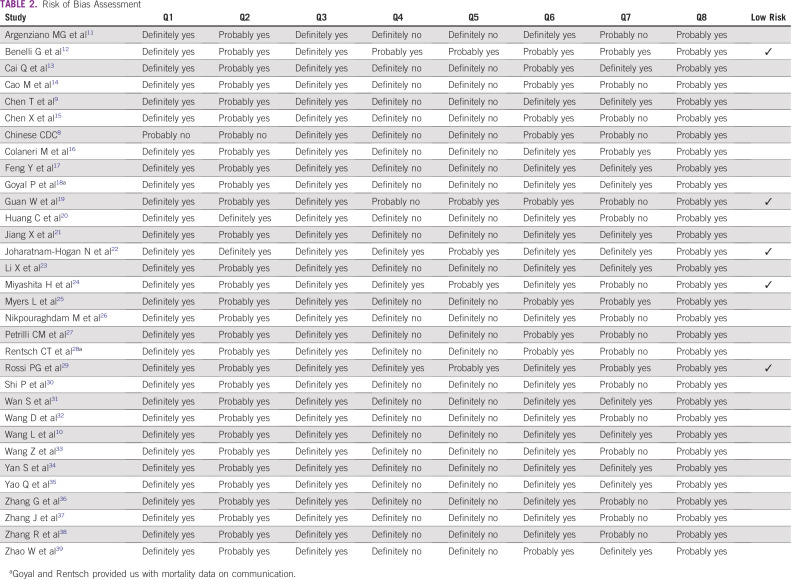
Risk of Bias Assessment

### All-Cause Mortality

**FIG 2:**
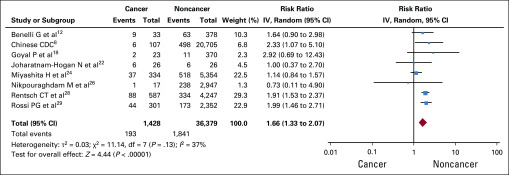
Forest plot for mortality. IV, inverse-variance.

Eight studies (37,807 total patients, 1,428 with cancer) provided data for all-cause mortality.^8,12,18,22,24,26,28,29^ No statistically significant heterogeneity was detected (*I*^2^ = 37%). All-cause mortality was higher in patients with versus without cancer (2,034 deaths; RR, 1.66; 95% CI, 1.33 to 2.07; *P* < .0001; Fig 2).

### Need for ICU Admission

**FIG 3:**
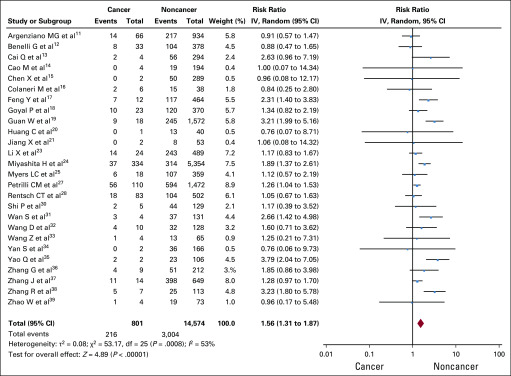
Forest plot for need for intensive care unit admission. IV, inverse-variance.

Twenty-six studies (15,375 total patients, 801 with cancer) provided data for need for ICU admission.^11-21,23-25,27,28,30-39^ Moderate significant heterogeneity was detected (*I*^2^ = 53%). Patients with cancer were more likely to need ICU admission than patients without cancer (3,220 events; RR, 1.56; 95% CI, 1.31 to 1.87; *P* < .0001; Fig 3).

### Sensitivity and Subgroup Analyses

In the sensitivity analysis of 4 studies with low risk of bias (8,804 total patients, 694 with cancer), all-cause mortality was higher in patients with versus without cancer (856 deaths; RR, 1.47; 95% CI, 1.04 to 2.09; *P* = .03).^12,22,24,29^ This was also the case for the sensitivity analyses by excluding each study and recalculating the RR.

**FIG 4:**
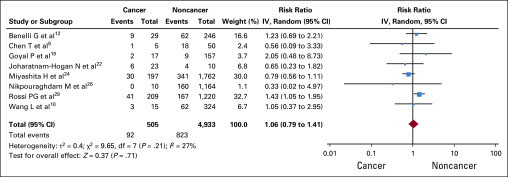
Forest plot for age subgroup analysis. IV, inverse-variance.

In the prespecified subgroup analysis of 8 studies (5,438 patients, 505 with cancer), which provided data on mortality of patients > 65 years old, all-cause mortality was comparable between those with versus without cancer (915 deaths; RR, 1.06; 95% CI, 0.79 to 1.41; *P* = .71; Fig 4).^9,10,12,18,22,24,26,29^

## DISCUSSION

By performing the most comprehensive meta-analysis to date, which incorporated data from > 46,000 patients with COVID-19 across almost all continents, we quantified the effect of cancer on all-cause mortality (RR, 1.66; 95% CI, 1.33 to 2.07) and need for ICU admission (RR, 1.56; 95% CI, 1.31 to 1.87). Also, by performing a prespecified subgroup analysis, we revealed the interesting finding that among patients > 65 years of age, all-cause mortality was comparable between those with versus without cancer.

It is important to quantify the effect of cancer on outcomes of patients with COVID-19, as there are conflicting reports in the literature. Several studies indicated that patients with cancer are more likely to develop severe disease and are at increased risk for poor prognosis.^40-42^ They therefore encouraged clinicians to treat patients with cancer as an extremely vulnerable population. Those studies might also raise issues as to whether it is futile to admit patients with cancer and COVID-19 to the ICU.^43^ On the other hand, other studies suggested that there was no evidence of elevated mortality rates among infected patients with cancer.^24,44^ An interesting theory even suggested that immunocompromised patients, such as patients with cancer, may dampen the so-called “cytokine storm” because of downregulated immune response and thus have comparable or even better clinical outcomes.^22,45^ The results of our meta-analysis might help to reveal the true effect of cancer on mortality and need for ICU admission.

An interesting finding of the meta-analysis was that, when data were collected from older patients, the increased mortality risk in the presence of cancer did not seem obvious. Regardless of cancer presence, increased age is considered a factor of worse prognosis.^46,47^ Furthermore, older individuals are characterized by an increased prevalence of comorbidities,^48^ which variably contribute to overall worse outcomes.^19^ On considering the aforementioned, the observed absence of increased mortality risk in older individuals does not conflict with the main findings of the study; it rather implies that the presence of cancer may not further affect the already burdened prognosis among individuals age > 65 years.

Our meta-analysis has limitations. First, there are concerns for duplicate publications,^4^ which might skew the results of any meta-analysis. In an attempt to minimize this risk, we excluded studies on mortality conducted in the same region with overlapping enrollment dates and we included only the results of the largest cohort. Second, data were not available to perform meaningful subgroup analyses by type of cancer (including treatment and immunosuppressive status). However, through communications with authors of original studies, we were able to carry out an important subgroup analysis by age.

In conclusion, by accumulating data from 32 studies involving 46,499 patients (1,776 patients with cancer) with COVID-19 from Asia, Europe, and the United States, we quantified the effect of cancer on important clinical outcomes, such as mortality and need for ICU admission. We also found that elderly patients with cancer may not be at increased risk of death when infected with COVID-19. The findings of the meta-analysis are important to clinicians, because they can inform discussions with patients about prognosis. They may also guide health policies regarding protection of this vulnerable population.
